# Seroprevalence and distribution of serogroup-specific pathogenic *Leptospira* antibodies in cattle and buffaloes in the state of Andhra Pradesh, India

**DOI:** 10.14202/vetworld.2019.1212-1217

**Published:** 2019-08-08

**Authors:** Anusha Alamuri, Sushma R. A. Thirumalesh, S. Sowjanya Kumari, K. Vinod Kumar, Parimal Roy, V. Balamurugan

**Affiliations:** 1 Indian Council of Agricultural Research-National Institute of Veterinary Epidemiology and Disease Informatics, Yelahanka, Bengaluru, Karnataka, India; 2 Department of Microbiology, Jain University, Bengaluru, Karnataka, India

**Keywords:** buffaloes, cattle, distribution of serovars, leptospirosis, microscopic agglutination test, seroprevalence

## Abstract

**Aim:**

In this study, the prevalence and the distribution status of *Leptospira* serogroup-specific antibodies among cattle and buffaloes in enzootic districts of Andhra Pradesh, a South Indian state was carried out.

**Materials and Methods:**

A total of 426 serum samples were randomly sampled from various villages from Prakasam, Kurnool, Guntur, Chittoor, Srikakulam, Visakhapatnam, and Godavari districts of Andhra Pradesh between 2016 and 2017. Serum samples from cattle (n=106) and buffaloes (n=320) having a history of pyrexia, and reproductive problems such as agalactia, infertility, abortions, and stillbirth. The serum samples were screened for *Leptospira*-specific antibodies by microscopic agglutination test using a reference panel of 18 live cultures of pathogenic *Leptospira* serovars.

**Results:**

The overall seropositivity of 68.08% (290/426) was observed with 70.8% (75/106) in cattle and 67.18% (215/320) in buffaloes. The frequency distribution of predominant serogroup-specific *Leptospira* antibodies in the sampled areas was determined against the employed serovars as follows: Icterohaemorrhagiae – 21.38%, Hebdomadis – 18.97%, Australis – 18.62%, Pomona – 17.24%, Sejroe – 15.86%, Tarassovi – 15.86%, Autumnalis – 15.52%, Panama – 14.83%, Shermani – 12.07%, Javanica – 11.38%, Hurstbridge – 11.03%, and Pyrogenes – 10.69%.

**Conclusion:**

It was evident that bovines had a role in maintaining several predominant *Leptospira* serovars with the change in the trend over a period. The results from this study would also help in strategizing and mitigating the disease burden in cattle and buffaloes of the enzootic area.

## Introduction

Leptospirosis is a global zoonosis caused by pathogenic strains of *Leptospires* [1]. The disease is neglected in most of the enzootic countries in the world due to lack of information and awareness of the extent of the public health problem [2]. The disease severity in humans and variety of mammalian hosts, such as rodents, cattle, buffaloes, sheep, goats, deer, pigs, dogs, camels, horses, raccoons, etc., impacts both public health and livestock economy [3]. The infection is usually transmitted by direct or indirect exposure to the skin (cuts/abrasions), mucous membranes (intact) to contaminated urine, placental fluids, etc. In cattle and other ruminants, leptospirosis accounts for great economic losses as a consequence of reduced milk yield (agalactia), abortion, stillbirth, reduced fertility, mortality in calves, decreased daily weight gain, the birth of weak calves, and mortality in calves [3,4]. *Leptospira* infection in bovines causes maintenance of bacteria in the host, leading to a carrier state. The incidence of cattle leptospirosis was first acknowledged in 1935 by Michin and Azinow. The Bernkopf isolated and established, *Leptospira* as the causative agent of disease in Palestine [5]. Since then, the disease is being reported globally, through infections by a wide variety of serovars, and with varied clinical outcomes [6].

Bovine leptospirosis has been sparsely reported in enzootic states of India. Seroprevalence of leptospirosis with various *Leptospira* serovars from farm animals in enzootic coastal states of India such as areas of Odisha, Maharashtra, Kerala, Tamil Nadu, Gujarat, and Andaman Islands [7-9] ranged from 14.55% [10] to 54.14% [11] has been reported. Further, a few studies have also been performed in urban areas, as leptospirosis has emerged as an important urban zoonosis [12]. The previous studies have reported seroprevalence of leptospirosis in Tamil Nadu [13], Kerala [14], Karnataka [15], and Andhra Pradesh [16]. However, the studies from Andhra Pradesh did not give the status on the breadth of circulating serovars as they were limited to only a few serovars [11,16,17]. Knowledge of prevalent *Leptospira* serovar(s) in a particular geographical area either in the incidental hosts or in carrier animals is essential to understand the epizootiology of the disease [18]; however, there are variations observed in the prevalence of serovars among different regions in a country or among different countries in the world. Moreover, it is often seen that bovine maintains several predominant *Leptospira* serovars with the change in the trend over a period.

Therefore, it is worthwhile to understand the prevalence and distribution of various serovars associated with leptospirosis over a period of time in specific geographical areas with the inclusion of more reference serovars in a test panel of microscopic agglutination test (MAT) including serovars of intermediate species [7]. This would help in understanding the linkage between circulating serovars in animals and humans as well as to apply strategies to mitigate the burden of leptospirosis. Since leptospirosis is a zoonosis, appropriate control measures can be adopted based on one health approach.

In this study, the prevalence and distribution of *Leptospira* serogroup-specific antibodies in cattle and buffaloes associated with a history of abortions and reproductive disorders, which are reared in rural areas (villages) of various districts, of Andhra Pradesh, India, were determined by employing a greater number of serovars in the antigen panel of MAT.

## Materials and Methods

### Ethical approval and Informed consents

Before sampling, the aim of the present study was explained to each farmer and the farmers who accepted to participate gave their oral consent before animal sampling. Moreover, samples were collected by well-trained veterinarians with respect regarding animal welfare regulations.

### Study area

Andhra Pradesh takes a coastline of 974 km (15.9129 N-79.7400 E) with the second extensive coastline among the states of India, after Gujarat. The study was conducted between 2016 and 2017 from different districts, namely, Chittoor, Guntur, Kurnool, Prakasam, Godavari, Srikakulam, and Visakhapatnam of Andhra Pradesh, where leptospirosis is enzootic.

### Serum samples

A total of 426 random serum samples from cattle and buffaloes were collected by local Veterinarians from apparently healthy animals with a history of fever, and reproductive disorders such as reduced milk yield (agalactia), abortion, stillbirth, and infertility from different villages in selected areas in various districts as stated above. Collected serum samples were transported on ice and stored at −20°C until further use.

### MAT

All the serum samples were screened for *Leptospira* serogroup-specific antibodies by MAT as described earlier at 1:100 dilution [19,20]. A panel of 18 pathogenic reference serovars, namely, Australis, Bankinang, Canicola, Hardjo, Hebdomadis, Tarassovi, Pyrogenes, Icterohaemorrhagiae, Pomona, Shermani, Kaup, Grippotyphosa, Hurstbridge, Javanica, Panama, Djasiman, Copenhageni, and Bataviae was included in the antigen panel while employing MAT [19]. A MAT titer of ≥1:100 was taken as a positive reactor as per the WHO/OIE manual for leptospirosis [20].

## Results and Discussion

The Indian state of *Andhra Pradesh* has a rich inheritance of livestock rearing [21]. The prevalence data with specific serovars of *Leptospira* infection in the coastal and non-coastal regions are mostly unknown in enzootic states of India. The local abundance of several species of pathogenic serovars may be a useful indicator to assess the potential transmission among animals and to humans, and it may help in taking appropriate control measures [22].

Further, there are no ample reports on the magnitude of *Leptospira* exposure in the bovine population, especially some of the enzootic coastal states of India; however, this study was undertaken to determine the seroprevalence of leptospirosis among the bovine population in rural areas of different districts of Andhra Pradesh with the distribution of serogroup-specific antibodies. However, in the past decade, there have been a few studies on the prevalence of leptospirosis in Andhra Pradesh by MAT using a limited panel of serovars (Bankinang, Icterohaemorrhagiae, Canicola, Hardjo, Hebdomadis, Grippotyphosa, Javanica, and Pomona) [16] and Linnodee ELISA Hardjo kit [8].

The overall seropositivity of 68.07% (290/426) was observed from different studied areas of Andhra Pradesh with seropositivity of 70.75% (75/106) in cattle and 67.18% (215/320) in buffaloes (Table-1), which was significantly higher than previously reported studies. This could be due to the samples collected from animals which were associated invariably with fever, abortion, and other reproductive problems and were suspected for leptospirosis. The results were in concordance with the earlier reported prevalence range from 45 to 70% while studying the bovine leptospirosis in enzootic states of India [16,19]. Balamurugan *et al*. [19] reported that seropositivity of 70.51% in cattle associated with reproductive problems in enzootic states of India, whereas Prameela *et al*. [16] observed the seropositivity of 44.73% with predominant serovar Pomona, Autumnalis, and Hardjo in cattle associated with pyrexia and abortions in the Andhra Pradesh, India.

**Table 1:** District wise details of serum samples screened by MAT and its test results for leptospirosis.

Andhra Pradesh	Buffaloes		Cattle		Total		Prevalent *Leptospira* serogroup specific antibodies	Number of reacted samples with *Leptospira* intermediate spp. serovars**
			
Districts	Number of samples tested	Number of samples showed positive reaction*	Number of samples tested	Number of samples showed positive reaction*	Number of samples tested	Number of samples showed positive reaction*		
Chittoor	1	0 (0)	23	17 (73.91)	24	17 (70.83)	Aus, Can, Sej, Heb, Pyr, Ict, Pom, She, Tar, Gri, Hus, Jav, Dja	Hus (6), Kau (2)
Guntur	85	60 (70.6)	3	2 (66.67)	88	62 (70.45)	Aus, Aut, Can, Sej, Heb, Pyr, Ict, Pom, She, Gri, Hus, Jav, Pan, Dja, Bat, Tar	Hus (6), Kau (10)
Kurnool	66	45 (68.19)	7	6 (85.71)	73	51 (69.86)	Aus, Aut, Can, Sej, Heb, Pyr, Ict, Pom, Gri, Hus, Jav, Pan, Bat, Tar	Hus (4), Kau (1)
Visakhapatnam	19	12 (63.15)	23	18 (78.26)	42	30 (71.43)	Aus, Aut, Can, Sej, Heb, Pyr, Ict, Pom, She, Tar, Gri, Hus, Jav, Pan, Dja, Bat	Hus (4), Kau (2)
Godavari	70	47 (67.15)	20	14 (70)	90	61 (67.78)	Aus, Aut, Can, Sej, Heb, Pyr, Ict, Pom, She, Tar, Gri, Hus, Jav, Pan, Dja, Bat	Hus (8), Kau (5)
Srikakulam	8	7 (87.5)	30	18 (60)	38	25 (65.79)	Aus, Aut, Can, Sej, Heb, Pyr, Ict, Pom, Tar, Gri, Hus, Jav, Pan, Dja, Bat	Hus (1), Kau (2)
Prakasam	71	44 (61.97)	0	0 (0)	71	44 (61.97)	Aus, Aut, Can, Sej, Heb, Pyr, Ict, Pom, She, Gri, Hus, Jav, Pan, Dja, Bat, Tar	Hus (3)
Total	320	215 (67.18)	106	75 (70.75)	426	290 (68.07)		

Aus=Australis, Aut=Autumnalis, Can=Canicola, Sej=Sejroe, Heb=Hebdomadis, Pyr=Pyrogenes, Ict=Icterohaemorrhagiae, Pom=Pomona, She=Shermani, Kau=Kaup, Gri=Grippotyphosa, Hus=Hurstbridge, Jav=Javanica, Pan=Panama, Dja=Djasiman, Tar=Tarassovi, Bat=Bataviae.

* Parenthesis indicates – % positivity,

** Parenthesis indicates – Number of reacted samples

Further on analysis, the highest seropositivity was observed in Visakhapatnam (71.43%), followed by Chittoor (70.83%), Guntur (70.45%), Kurnool (69.86%), Godavari (67.78%), Srikakulam (65.79%), and Prakasam (61.97%) districts. According to the previous studies, high prevalence is often observed in the coastal regions compared to the non-coastal regions. Andhra Pradesh is a coastal state with most of its districts lying in the coastline such as Visakhapatnam, Srikakulam, and Prakasam. However, there was not much difference found in coastal and non-coastal districts, but the prevalence in the non-coastal districts (70.27% [130/185]) was slightly higher compared to the coastal districts (66.39% [160/241]) (Table-1). Whether it is a coastal or non-coastal region, the observed rearing conditions such as open herds, using shared bulls for breeding, and shared grazing with common water sources are almost similar in both the regions as there is a chance of getting the *Leptospira* infection by rearing conditions and management practices.

The frequency distribution of pathogenic *Leptospira* antibodies was determined against various serovars employed as shown in Table-2. The major reacted prevalent pathogenic serovars (frequency distribution from 19 to 12%) representing serogroup antibodies in different districts were Icterohaemorrhagiae, Hebdomadis, Australis, Pomona, Sejroe, Tarassovi, Autumnalis, Panama, Shermani, etc. Further, considering the serovars status, the predominant serovars pattern is similar to the studies conducted earlier by Balakrishnan *et al*. [11] and Prameela *et al*. [16] except that the serovars Australis and Hebdomadis observed in the present study along with other emerging pathogens of different serovars, as the change in trend of prevalent predominant serovars over a period of time noticed [23,24].

**Table 2:** Frequency percentage distribution of reacted pathogenic serovars in cattle and buffaloes in endemic districts of Andhra Pradesh.

*Leptospira* serovars	Overall sample reactivity	Frequency percentage reactivity (%)
Hebdomadis	55	18.97
Australis	54	18.62
Pomona	50	17.24
Hardjo	46	15.86
Tarassovi	46	15.86
Bankinang	45	15.52
Panama	43	14.83
Icterohaemorrhagiae	36	12.41
Shermani	35	12.07
Javanica	33	11.38
Hurstbridge	32	11.03
Pyrogenes	31	10.69
Copenhageni	30	10.34
Bataviae	29	10.00
Kaup	22	7.59
Canicola	21	7.24
Djasiman	20	6.90
Grippotyphosa	16	5.52

Although the serovar Hardjo is considered as common in cattle [25], other serovars are also prominent in many parts of the world. Moreover, the previous study conducted during 2008 in different districts of Andhra Pradesh showed 67.15% (92/137) in cattle and 61.9% (52/84) in buffaloes associated with reproductive problems with Pomona, Pyrogenes, Hebdomadis, Hardjo, Australis, etc., as predominant serovars [17]. Further, during the year 2011, Balakrishnan *et al*. [11] reported 50.21% (122/243) in cattle and 68.64% (90/118) in buffaloes with Hebdomadis, Pomona, Ballum, Hardjo, etc., as predominant serovars, whereas Prameela *et al*. [16] reported 44.73% (17/38) prevalence with 35% Pomona, 17% Autumnalis, and 11.76% Hardjo as predominant serovars in cattle associated with pyrexia and abortions.

Further, the number of seropositive samples to intermediate and other *Leptospira* spp. serovars evidenced (Table-1), the re-emergence of *Leptospira* pathogens. Our earlier study indicates the prevalence of intermediate species serovars in ruminants in enzootic states of India [7], especially the emergence of Kaup and Hurstbridge serovars representing *L. inadai* and *L. fainei* species for bovine samples. Therefore, by employing 18 reference serovars panel in MAT, as against eight serovars employed in the earlier study [16], we could able to detect a greater number of seroreactive animals. The additional seropositivity of 15.02% (64/426) was observed while including other serovars in the test panels, which are not covered in the earlier study [16]. Further, a total of 10 bovine samples reacted (3.44%) exclusively to Kaup (3) and Hurstbridge (7) with *Leptospira* intermediate spp. serovars.

The observed cross-reactivity between different serovars in cattle and buffaloes is presented in Table-3, with the major selected reacted reference serovars versus cross-reactive serovars are summarized in Table-4. Overall, with nine selected major reacted serovars (Hebdomadis, Australis, Pomona, Hardjo, Tarassovi, Autumnalis, Panama, Icterohaemorrhagiae, and Shermani) in the panel, it is possible to detect the *Leptospira* antibodies in bovine serum samples for providing diagnosis more than 82%, in MAT with coverage ranges from 86 to 92% in some districts (Kurnool, Visakhapatnam, Prakasam, and Guntur) of Andhra Pradesh. Further, with the inclusion of two intermediate serovars, it is possible to cover overall 85% of the positive MAT reacted samples. However, in this study, as the samples were limited to a few villages in some districts, it is recommended that further testing be done using large sample size. In a nutshell, there was a significant change observed in the trend of prevalent predominant serovars in this particular geographical area over a period of time with the emergence of Hebdomadis, Australis, Panama, and Shermani including intermediate species serovars Kaup and Hurstbridge, etc.

**Table 3:** Cross-reactivity among different *Leptospira* serovars in cattle and buffaloes in endemic districts of Andhra Pradesh.

Serovars	Aus	Ban	Can	Har	Heb	Pyr	Tar	Ict	Pom	She	Kau	Gri	Hus	Jav	Pan	Dja	Cop	Bat
Aus	-	14	3	2	12	3	11	5	10	2	3	1	8	1	4	1	1	3
Ban	-	-	0	1	5	12	6	3	9	2	1	1	2	5	3	4	2	4
Can	-	-	-	3	1	3	0	2	0	1	3	4	1	0	4	1	1	2
Har	-	-	-	-	2	4	0	1	2	1	0	2	4	0	3	0	2	1
Heb	-	-	-	-	-	4	19	6	15	6	5	3	7	10	8	1	8	4
Pyr	-	-	-	-	-	-	1	0	6	1	1	2	5	4	2	3	2	2
Tar	-	-	-	-	-	-	-	1	17	15	9	2	7	12	3	1	5	4
Ict	-	-	-	-	-	-	-	-	4	2	2	4	2	3	7	5	4	6
Pom	-	-	-	-	-	-	-	-	-	17	10	1	3	11	6	1	1	1
She	-	-	-	-	-	-	-	-	-	-	9	1	6	11	5	2	3	1
Kau	-	-	-	-	-	-	-	-	-	-	-	1	3	8	1	0	0	3
Gri	-	-	-	-	-	-	-	-	-	-	-	-	3	1	3	0	3	2
Hus	-	-	-	-	-	-	-	-	-	-	-	-	-	5	3	0	4	2
Jav	-	-	-	-	-	-	-	-	-	-	-	-	-	-	7	2	4	1
Pan	-	-	-	-	-	-	-	-	-	-	-	-	-	-	-	5	3	3
Dja	-	-	-	-	-	-	-	-	-	-	-	-	-	-	-	-	2	0
Cop	-	-	-	-	-	-	-	-	-	-	-	-	-	-	-	-	-	2
Bat	-	-	-	-	-	-	-	-	-	-	-	-	-	-	-	-	-	-

Aus=Australis, Ban=Bankinang, Can=Canicola, Har=Hardjo, Heb=Hebdomadis, Pyr=Pyrogenes, Ict=Icterohaemorrhagiae, Pom=Pomona, She=Shermani, Kau=Kaup, Gri=Grippotyphosa, Hus=Hurstbridge, Jav=Javanica, Pan=Panama, Dja=Djasiman, Cop=Copenhageni, Bat=Bataviae, Tar=Tarassovi

**Table 4:** Majorly selected reacted serovars versus cross-reacted serovars.

Major selected serovars	Cross-reacted serovars
Hebdomadis	Tar, Pom, Aus, Jav, Har, Pan, Cop, Hus, Ict, She, Ban, Kau, Pyr, Bat, Gri, Can, Dja
Australis	Heb, Ban, Tar, Hus, Pom, Har, Ict, Pan, Can, Pyr, Kau, Bat, She, Gri, Jav, Dja, Cop
Pomona	Tar, She, Heb, Har, Jav, Kau, Aus, Ban, Pyr, Pan, Ict, Hus, Gri, Dja, Cop, Bat, Can
Hardjo	Pom, Heb, Ban, Pyr, She, Jav, Aus, Tar, Hus, Pan, Can, Cop, Gri, Ict, Bat, Kau
Tarassovi	Heb, Pom, She, Jav, Aus, Kau, Hus, Ban, Cop, Har, Bat, Pan, Gri, Pyr, Ict
Bankinang	Pyr, Aus, Har, Tar, Pom, Heb, Jav, Dja, Bat, Pan, Ict, She, Hus, Cop, Kau, Gri
Panama	Heb, Ict, Jav, Pom, She, Dja, Aus, Can, Har, Ban, Tar, Gri, Hus, Cop, Bat, Pyr
Icterohaemorrhagiae	Pan, Heb, Bat, Dja, Aus, Gri, Cop, Pom, Jav, Ban, Can, She, Kau, Hus, Har, Tar, Pyr
Shermani	Pom, Tar, Jav, Kau, Heb, Hus, Har, Pan, Cop, Aus, Ban, Ict, Dja, Can, Pyr, Gri, Bat
Total cross-reacted serovars	Heb, Tar, Pom, Aus, Jav, Har, Pan, Cop, Hus, Ict, She, Ban, Kau, Pyr, Bat, Gri, Can, Dja

Aus=Australis, Ban=Bankinang, Can=Canicola, Heb=Hebdomadis, Pyr=Pyrogenes, Tar=Tarassovi Ict=Icterohaemorrhagiae, Pom=Pomona, She=Shermani, Kau=Kaup, Gri=Grippotyphosa, Hus=Hurstbridge, Jav=Javanica, Pan=Panama, Dja=Djasiman, Cop=Copenhageni, Bat=Bataviae, Har=Hardjo

## Conclusion

The seroprevalence of leptospirosis among cattle and buffaloes in different districts of Andhra Pradesh was significantly high and bovine has a role in maintaining several predominant serovars with the change in trend over a period of time. The continuous presence of these *Leptospires* will lead to a potential zoonotic risk to slaughterhouse workers, meat inspectors, animal holders, agriculture laborers, farmers, etc., which also determines the need for continuous monitoring of leptospirosis in animals and in humans to combat this zoonosis. Although there have been studies conducted in different parts of India, it is difficult to assess the true nature of the disease in epizootiological point, due to inadequate sample size. Thus, intensive surveillance programs, preventive vaccinations, and control strategies to reduce the incidence or burden of bovine leptospirosis are warranted, which, in turn, help in reducing the zoonotic infections to various stakeholders including public health personnel.

## Authors’ Contributions

The present study is the part of AA’s Ph.D., dissertation. AA visited the field, collected the samples and drafted the manuscript. AA, SRAT, and SSK carried out the laboratory experiment, KVK analyzed the data and edited the manuscript. PR provided guidance and support to carry out the experiments. VB designed the work with overall monitoring, analyzed the data and edited the manuscript. All authors drafted, read, and approved the final manuscript.

## References

[1] Vijayachari P, Sugunan A, Shriram A (2008). Leptospirosis:An emerging global public health problem. J. Biosci.

[2] Srivastava S.K (2008). Current status of leptospirosis in India in animals and humans. Indian J. Vet. Pathol.

[3] Ellis W.A (2015). Animal leptospirosis. Curr. Top. Microbiol. Immunol.

[4] Thiermann A.B (1984). Leptospirosis:Current developments and trends. J. Am. Vet. Med. Assoc.

[5] Smith D.L.T, Perry D.A (1952). Bovine leptospirosis case histories. Can. J. Comp. Med.

[6] Faine S, Adler B, Bolin C, Perolat P (1999). *Leptospira* and Leptospirosis.

[7] Balamurugan V, Thirumalesh S.R.A, Sridevi R, Govindaraj G, Nagalingam M, Hemadri D, Gajendragad M.R, Rahman H (2016). Microscopic agglutination test analysis identifies prevalence of intermediate species serovars in ruminants in endemic states of India. Proc. Natl. Acad. Sci. India B Biol. Sci.

[8] Balamurugan V, Alamuri A, Veena S, Bharathkumar K, Patil S.S, Govindaraj G, Rahman H (2016). Investigation on the prevalence of *Leptospira* serovar Hardjo in organized cattle dairy farms of India. Indian J. Anim. Sci.

[9] Sharma S, Vijayachari P, Sugunan A.P, Sehgal S.C (2003). Leptospiral carrier state and seroprevalence among animal population-a cross-sectional sample survey in Andaman and Nicobar Islands. Epidemiol. Infect.

[10] Savalia C.V (2001). Seroprevalence of leptospirosis in bovines of Gujarat. Veterinar.

[11] Balakrishnan G, Roy P, Govindarajan R, Ramaswamy V, Manohar M.B (2011). Bovine leptospirosis in Andhra Pradesh. Indian Vet. J.

[12] Hotez P.J (2017). Global urbanization and the neglected tropical diseases. PLoS Negl. Trop. Dis.

[13] Koteeswaran A (2006). Seroprevalence of leptospirosis in man and animals in Tamilnadu. Indian J. Med. Microbiol.

[14] Adinarayanan N, James P.C (1980). Studies on leptospirosis in farm stock and wildlife in Kerala. Isolation of leptospires from divergent classes of animal hosts with an outbreak of cultural procedure. Indian Vet. J.

[15] Upadhye A.S (1982). Leptospirosis an etiological agent of abortion in bovines. Indian J. Comp. Microbiol. Immunol. Infect. Dis.

[16] Prameela R.D, Sreenivasulu D, Vijayachari P, Seenivasan N.N (2013). Seroepidemiology of leptospirosis in Andhra Pradesh. Arch. Clin. Microbiol.

[17] Balakrishnan G, Govindarajan R, Meenambigai T.V, Jayakumar V, Manohar B.M (2008). Seroprevalence of leptospirosis among domestic animals in Andhra Pradesh. Indian Vet. J.

[18] Balamurugan V, Veena S, Thirumalesh S.R.A, Alamuri A, Sridevi R, Sengupta P.P, Govindaraj G, Nagalingam M, Hemadri D, Gajendragad M.R, Rahman H (2017). Distribution of serogroup specific antibodies against leptospirosis in livestock in Odisha. Indian J. Anim. Sci.

[19] Balamurugan V, Alamuri A, Bharathkumar K, Patil S.S, Govindaraj G.N, Nagalingam M, Krishnamoorthy P, Rahman H, Shome B.R (2018). Prevalence of *Leptospira* serogroup-specific antibodies in cattle associated with reproductive problems in endemic states of India. Trop. Anim. Health Prod.

[20] OIE (2013). Office International des Épizooties (OIE-World Organisation for Animal Health). Manual of Diagnostic Tests and Vaccines for Terrestrial Animals.

[21] Kishore K (2019). Livestock Rearing in the Indian Context LEISA.

[22] Chadsuthi S, Bicout D.J, Wiratsudakul A, Suwancharoen D, Petkanchanapong W, Modchang C, Triampo W, Ratanakorn P, Chalvet-Monfray K (2017). Investigation on predominant *Leptospira* serovars and its distribution in humans and livestock in Thailand, 2010-2015. PLoS Negl. Trop. Dis.

[23] Lall C, Kumar K.V, Raj R.V, Sugunan A.P, Sunish I.P, Sharma S, Vijayachari P (2017). Trend in the seroprevalence of Leptospirosis among cattle and goat populations of South Andaman. Indian J. Vet Res.

[24] Raj R.V, Kumar K.V, Lall C, Vedhagiri K, Sugunan A.P, Sunish I.P, Sharma S, Vijayachari P (2018). Changing trend in the seroprevalence and risk factors of human leptospirosis in the South Andaman Island, India. Zoonoses Public Health.

[25] Monte L.G, Ridieri K.F, Jorge S, Oliveira N.R, Hartwig D.D, Amaral M.G, Hartleben C.P, Dellagostin O.A (2015). Immunological and molecular characterization of *Leptospira interrogans* isolated from a bovine fetus. Comp. Immunol. Microbiol. Infect. Dis.

